# Supplementary figures and images for: Improved genome of Agrobacterium radiobacter type strain provides new taxonomic insight into Agrobacterium genomospecies 4
Source: PeerJ. 2019 Feb 8;7:e6366. doi: 10.7717/peerj.6366 (PMC6369824; doi:10.7717/peerj.6366)

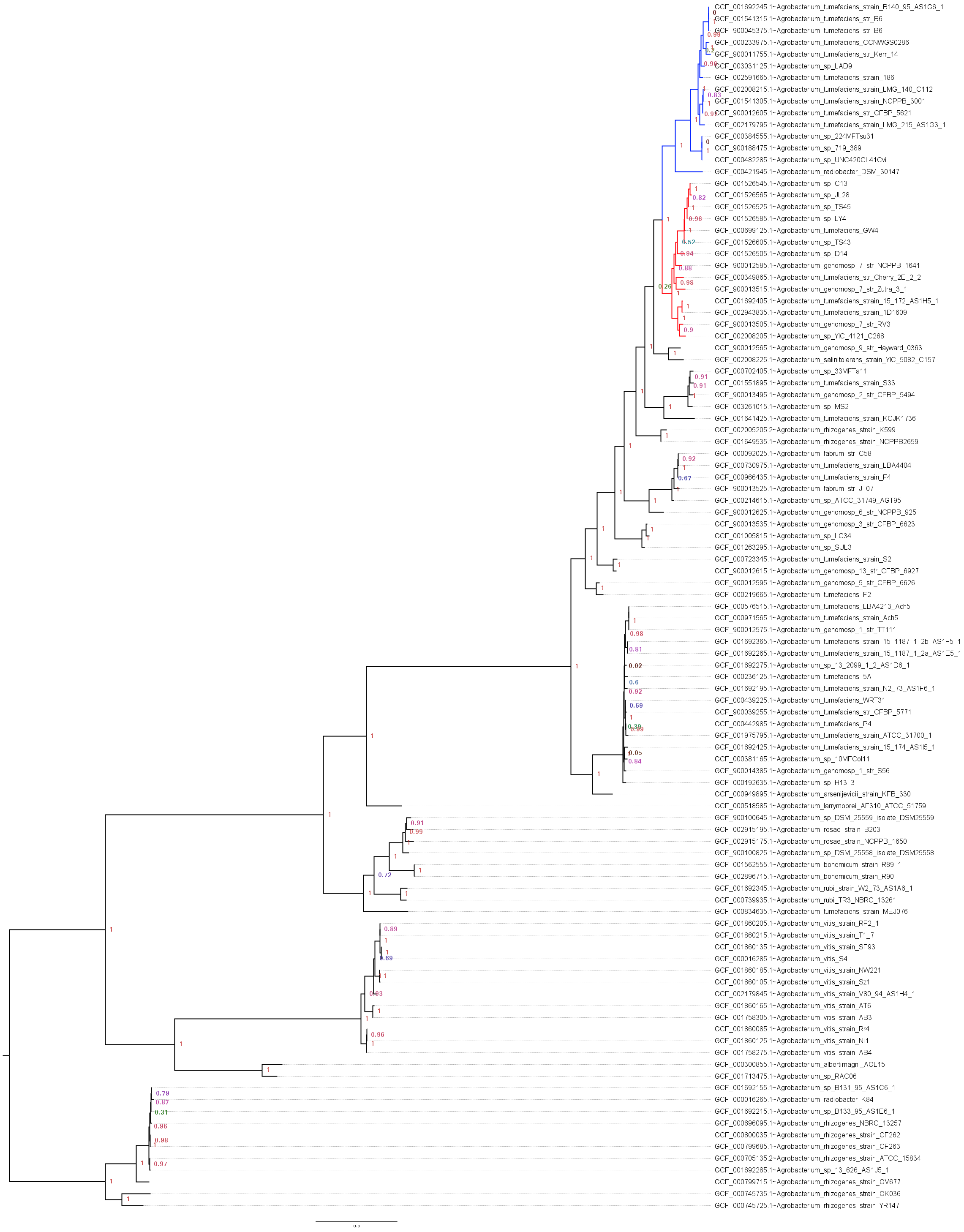

Supplement: Supplemental Information 1 — The tree was rooted with members from the species Rhizobium rhizogenes (labeled as Agrobacterium rhizogenes) as the outgroup. Blue and red-colored clades belong to Agrobacterium genomospecies 4 and 7, respectively. Node labels indicate local SH-like support values. Branch lengths indicate the number of substitutions per site. [file peerj-07-6366-s001.png]
