# Supplementary material for: Improved genome of Agrobacterium radiobacter type strain provides new taxonomic insight into Agrobacterium genomospecies 4
Source: PeerJ. 2019 Feb 8;7:e6366. doi: 10.7717/peerj.6366 (PMC6369824; doi:10.7717/peerj.6366)
Supplement: Supplemental Information 2 [file peerj-07-6366-s002.docx]

**Adapter Trimming (no quality trimming, read will be corrected with Spades) :**

java –jar trimmomatic-0.36.jar PE –phred33 Read1.fastq.gz Read2.fastq.gz Read1_trim.fastq.gz Read1_single.fastq.gz Read2_trim.fastq.gz Read2_single.fastq.gz ILLUMINACLIP:NexteraPE-PE.fa:2:30:10

***Read1.fastq.gz = File containing raw Read1 sequences***

***Read2.fastq.gz = File containing raw Read1 sequences***

***Read1_trim.fastq.gz = Adapter-trimmed sequences for Read1(Paired)***

***Read1_single.fastq.gz = Adapter-trimmed sequences Read1 (Unpaired)***

***Read2_trim.fastq.gz = Adapter-trimmed sequences for Read2(Paired)***

***Read2_single.fastq.gz = Adapter-trimmed sequences Read2 (Unpaired)***

***NexteraPE-PE.fa = Nextera Adapter sequences provided by Trimmomatic. Ensure that this file is soft-linked in the working directory.***

**Genome Assembly (Spades)**

Spades.py -1 Read1_trim.fastq.gz -2 Read2_trim.fastq.gz –o OutputFolder –careful –cov_cutoff auto

“—careful” = reduce number of mismatches and short indels by mapping the reads back to the assembled genome and correcting based on mapping quality

“--cov_cutoff auto” = automatic removal of low coverage contigs
